# 2-(2-Oxothio­lan-3-yl)isoindoline-1,3-dione

**DOI:** 10.1107/S1600536810043400

**Published:** 2010-10-30

**Authors:** Abdul Rauf Raza, Aisha Saddiqa, M. Nawaz Tahir, Sadia Saddiq

**Affiliations:** a Department of Chemistry, University of Sargodha, Sargodha, Pakistan; b Department of Physics, University of Sargodha, Sargodha, Pakistan

## Abstract

In the title compound, C_12_H_9_NO_3_S, the isoindoline-1,3-dione group is almost planar, with an r.m.s. deviation of 0.020 Å, whereas the heterocyclic ring approximates to an envelope with the methyl­ene group not adjacent to the S atom in the flap position. A short intra­molecular C—H⋯O contact generates an *S*(6) ring motif. In the crystal structure, weak aromatic π–π stacking inter­actions occur between the centroids of the benzene rings at a distance of 3.558 (2) Å.

## Related literature

For background to isocoumarins, see: Hussain *et al.* (2001 ▶); Lee *et al.* (2001 ▶); Nozawa *et al.* (1981 ▶). For related crystal structures, see: Beck *et al.* (2007 ▶); Freer & Kraut (1965 ▶). For graph-set notation, see: Bernstein *et al.* (1995 ▶).
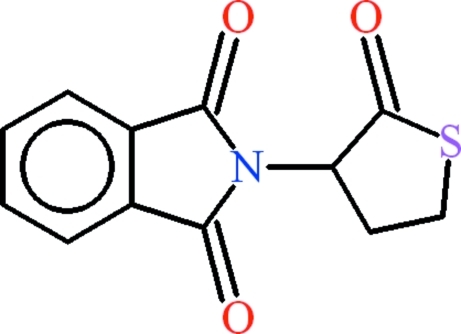


## Experimental

### 

#### Crystal data

C_12_H_9_NO_3_S
*M* *_r_* = 247.26
Monoclinic, 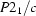

*a* = 8.0601 (13) Å
*b* = 6.9860 (11) Å
*c* = 19.709 (3) Å
β = 99.296 (9)°
*V* = 1095.2 (3) Å^3^
*Z* = 4
Mo *K*α radiation
μ = 0.29 mm^−1^
*T* = 296 K
0.24 × 0.10 × 0.08 mm

#### Data collection

Bruker Kappa APEXII CCD diffractometer
Absorption correction: multi-scan (*SADABS*; Bruker, 2005 ▶) *T*_min_ = 0.968, *T*_max_ = 0.978
14781 measured reflections
1934 independent reflections
1105 reflections with *I* > 2σ(*I*)
*R*_int_ = 0.093

#### Refinement

*R*[*F* ^2^ > 2σ(*F* ^2^)] = 0.055
*wR*(*F* ^2^) = 0.151
*S* = 1.02
1934 reflections
154 parameters
H-atom parameters constrained
Δρ_max_ = 0.40 e Å^−3^
Δρ_min_ = −0.31 e Å^−3^

### 

Data collection: *APEX2* (Bruker, 2009 ▶); cell refinement: *SAINT* (Bruker, 2009 ▶); data reduction: *SAINT*; program(s) used to solve structure: *SHELXS97* (Sheldrick, 2008 ▶); program(s) used to refine structure: *SHELXL97* (Sheldrick, 2008 ▶); molecular graphics: *ORTEP-3* (Farrugia, 1997 ▶) and *PLATON* (Spek, 2009 ▶); software used to prepare material for publication: *WinGX* (Farrugia, 1999 ▶) and *PLATON*.

## Supplementary Material

Crystal structure: contains datablocks global, I. DOI: 10.1107/S1600536810043400/hb5705sup1.cif

Structure factors: contains datablocks I. DOI: 10.1107/S1600536810043400/hb5705Isup2.hkl

Additional supplementary materials:  crystallographic information; 3D view; checkCIF report

## Figures and Tables

**Table 1:** Hydrogen-bond geometry (Å, °)

*D*—H⋯*A*	*D*—H	H⋯*A*	*D*⋯*A*	*D*—H⋯*A*
C10—H10*B*⋯O1	0.97	2.52	3.149 (5)	122

**Fig. 1.**
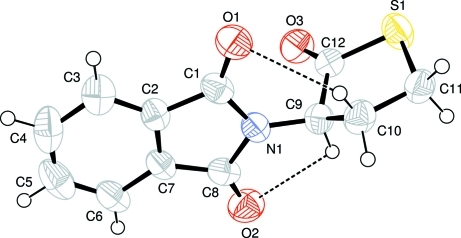
View of (I) with displacement ellipsoids drawn at the 50% probability level.

## supplementary crystallographic information

### Comment

Isocoumarins are important class of naturally occurring compounds. Their structure is similar to coumarin but with inverted lactone ring. They exhibit a wide range of biological activities such as antimicrobial (Hussain *et al.*, 2001), anti-fungal (Nozawa *et al.*, 1981), anti-angiogenic (Lee *et al.*, 2001). The title compound (I, Fig. 1) was obtained as an interesting side-product during the synthesis of isocoumarin.

The crystal structure of D,*L*-homocysteine thiolactone hydrochloride (Freer & Kraut, 1965) and (*R**)-2-(4-Chlorophenyl)-N-(hept-4-yl)-2-((S*)-2- oxotetrahydrothiophen-3-ylamino)acetamide (Beck *et al.*, 2007) have been reported which contain the heterocyclic ring.

The title compound essentially consists of monomers. In (I), the 2-benzoazole-1,3-dione group (C1–C8/N1/O1/O2) is planar with r.m.s. deviation of 0.0196 Å. The heterocyclic ring (C9/C10/C11/S1/C12) is not planar as the r.m.s. deviation of the plane is 0.1511 Å. The dihedral angle between these two groups is 88.05 (10)°. There exist weak intramolecular H-bondings of C—H···O type (Table 1, Fig. 1) completing S(5) and S(6) ring motifs (Bernstein *et al.*, 1995). There exist π–π interaction between the centroids of benzene rings at a distance of 3.558 (2) Å [symmetry: 1 - *x*, - *y*, - *z*].

### Experimental

Homophthallic acid (1.0 g, 5.5 mmol, 1 eq) was added to 4-(methylthio)-2-(1,3-dioxoisoindolin-2-yl)butanoyl chloride (6.5 g, 0.022 mol, 4 eq) and the mixture was heated at 473 K with continuous stirring for 6 h. The crude product was added to chilled water (20 ml), partitioned with EtOAc (3 × 25 ml), organic layer was dried over anhydrous Na_2_SO_4_, filtered and concentrated under reduced pressure. The column chromatographic separation using EtOAc and n-hexane (3:7) as mobile phase afforded the title compound (I) as colourless needles.

### Refinement

The H atoms were positioned geometrically with C—H = 0.93–0.97 Å and were included in the refinement in the riding model approximation, with *U*_iso_(H) = *xU*_eq_(C), where *x* = 1.2 for all H atoms.

### Crystal data

**Table:**

C_12_H_9_NO_3_S	*F*(000) = 512
*M**_r_* = 247.26	*D*_x_ = 1.500 Mg m^−^^3^
Monoclinic, *P*2_1_/*c*	Mo *K*α radiation, λ = 0.71073 Å
Hall symbol: -P 2ybc	Cell parameters from 1105 reflections
*a* = 8.0601 (13) Å	θ = 2.1–25.0°
*b* = 6.9860 (11) Å	µ = 0.29 mm^−^^1^
*c* = 19.709 (3) Å	*T* = 296 K
β = 99.296 (9)°	Needle, colourless
*V* = 1095.2 (3) Å^3^	0.24 × 0.10 × 0.08 mm
*Z* = 4	

### Data collection

**Table:**

Bruker Kappa APEXII CCD diffractometer	1934 independent reflections
Radiation source: fine-focus sealed tube	1105 reflections with *I* > 2σ(*I*)
graphite	*R*_int_ = 0.093
Detector resolution: 8.2 pixels mm^-1^	θ_max_ = 25.0°, θ_min_ = 2.1°
ω scans	*h* = −9→9
Absorption correction: multi-scan (*SADABS*; Bruker, 2005)	*k* = −8→8
*T*_min_ = 0.968, *T*_max_ = 0.978	*l* = −23→23
14781 measured reflections	

### Refinement

**Table:**

Refinement on *F*^2^	Primary atom site location: structure-invariant direct methods
Least-squares matrix: full	Secondary atom site location: difference Fourier map
*R*[*F*^2^ > 2σ(*F*^2^)] = 0.055	Hydrogen site location: inferred from neighbouring sites
*wR*(*F*^2^) = 0.151	H-atom parameters constrained
*S* = 1.02	*w* = 1/[σ^2^(*F*_o_^2^) + (0.0651*P*)^2^ + 0.3208*P*] where *P* = (*F*_o_^2^ + 2*F*_c_^2^)/3
1934 reflections	(Δ/σ)_max_ < 0.001
154 parameters	Δρ_max_ = 0.40 e Å^−^^3^
0 restraints	Δρ_min_ = −0.31 e Å^−^^3^

### Special details

**Table:**

Geometry. Bond distances, angles *etc*. have been calculated using the rounded fractional coordinates. All su's are estimated from the variances of the (full) variance-covariance matrix. The cell e.s.d.'s are taken into account in the estimation of distances, angles and torsion angles
Refinement. Refinement of *F*^2^ against ALL reflections. The weighted *R*-factor *wR* and goodness of fit *S* are based on *F*^2^, conventional *R*-factors *R* are based on *F*, with *F* set to zero for negative *F*^2^. The threshold expression of *F*^2^ > σ(*F*^2^) is used only for calculating *R*-factors(gt) *etc*. and is not relevant to the choice of reflections for refinement. *R*-factors based on *F*^2^ are statistically about twice as large as those based on *F*, and *R*- factors based on ALL data will be even larger.

### Fractional atomic coordinates and isotropic or equivalent isotropic displacement parameters (Å^2^)

**Table:**

	*x*	*y*	*z*	*U*_iso_*/*U*_eq_	
S1	1.27260 (14)	0.17317 (15)	0.21281 (6)	0.0666 (4)	
O1	1.0043 (4)	0.2417 (4)	0.01824 (15)	0.0690 (11)	
O2	0.6135 (4)	0.3134 (4)	0.15927 (16)	0.0694 (11)	
O3	1.0050 (3)	−0.0304 (4)	0.16664 (14)	0.0619 (11)	
N1	0.8391 (4)	0.2932 (4)	0.10189 (16)	0.0463 (11)	
C1	0.8677 (5)	0.2558 (5)	0.0348 (2)	0.0496 (14)	
C2	0.6994 (5)	0.2417 (5)	−0.0077 (2)	0.0445 (14)	
C3	0.6538 (6)	0.2073 (5)	−0.0763 (2)	0.0600 (16)	
C4	0.4821 (7)	0.1996 (6)	−0.1015 (2)	0.0724 (19)	
C5	0.3638 (6)	0.2223 (6)	−0.0585 (3)	0.073 (2)	
C6	0.4108 (5)	0.2546 (5)	0.0105 (3)	0.0580 (16)	
C7	0.5801 (4)	0.2646 (5)	0.0350 (2)	0.0469 (14)	
C8	0.6676 (5)	0.2926 (5)	0.1061 (2)	0.0486 (14)	
C9	0.9686 (4)	0.3132 (5)	0.1613 (2)	0.0492 (12)	
C10	1.0927 (5)	0.4715 (5)	0.1568 (2)	0.0537 (16)	
C11	1.2487 (5)	0.4309 (5)	0.2093 (2)	0.0553 (16)	
C12	1.0662 (5)	0.1259 (5)	0.17663 (18)	0.0444 (12)	
H3	0.73362	0.18977	−0.10491	0.0718*	
H4	0.44640	0.17879	−0.14821	0.0870*	
H5	0.25006	0.21559	−0.07677	0.0880*	
H6	0.33150	0.26915	0.03956	0.0696*	
H9	0.91266	0.34070	0.20085	0.0591*	
H10A	1.04437	0.59374	0.16649	0.0646*	
H10B	1.12157	0.47652	0.11092	0.0646*	
H11A	1.23521	0.48081	0.25402	0.0667*	
H11B	1.34670	0.49028	0.19538	0.0667*	

### Atomic displacement parameters (Å^2^)

**Table:**

	*U*^11^	*U*^22^	*U*^33^	*U*^12^	*U*^13^	*U*^23^
S1	0.0529 (7)	0.0572 (7)	0.0828 (9)	0.0032 (5)	−0.0094 (6)	0.0022 (6)
O1	0.0456 (18)	0.086 (2)	0.078 (2)	−0.0061 (15)	0.0178 (16)	−0.0142 (16)
O2	0.0619 (19)	0.075 (2)	0.076 (2)	−0.0060 (16)	0.0255 (17)	−0.0102 (16)
O3	0.0674 (19)	0.0400 (16)	0.078 (2)	−0.0076 (14)	0.0106 (16)	−0.0004 (14)
N1	0.0420 (19)	0.0466 (18)	0.049 (2)	0.0011 (14)	0.0038 (16)	0.0021 (15)
C1	0.040 (2)	0.042 (2)	0.067 (3)	−0.0027 (18)	0.009 (2)	0.0031 (18)
C2	0.043 (2)	0.037 (2)	0.052 (3)	−0.0082 (16)	0.003 (2)	0.0049 (17)
C3	0.069 (3)	0.051 (2)	0.058 (3)	−0.013 (2)	0.004 (2)	0.009 (2)
C4	0.091 (4)	0.056 (3)	0.059 (3)	−0.017 (3)	−0.022 (3)	0.010 (2)
C5	0.054 (3)	0.044 (3)	0.111 (5)	−0.006 (2)	−0.019 (3)	0.018 (3)
C6	0.041 (2)	0.037 (2)	0.092 (4)	−0.0023 (18)	−0.001 (2)	0.004 (2)
C7	0.039 (2)	0.029 (2)	0.069 (3)	−0.0025 (16)	−0.002 (2)	0.0051 (17)
C8	0.042 (2)	0.038 (2)	0.068 (3)	−0.0018 (17)	0.016 (2)	0.0010 (19)
C9	0.046 (2)	0.048 (2)	0.053 (2)	−0.0043 (18)	0.006 (2)	−0.0034 (19)
C10	0.061 (3)	0.041 (2)	0.058 (3)	−0.005 (2)	0.006 (2)	−0.0018 (18)
C11	0.056 (3)	0.053 (2)	0.056 (3)	−0.007 (2)	0.006 (2)	−0.009 (2)
C12	0.048 (2)	0.042 (2)	0.044 (2)	−0.0006 (18)	0.0098 (19)	0.0016 (17)

### Geometric parameters (Å, °)

**Table:**

S1—C11	1.811 (4)	C6—C7	1.374 (6)
S1—C12	1.733 (4)	C7—C8	1.477 (5)
O1—C1	1.202 (5)	C9—C10	1.503 (5)
O2—C8	1.207 (5)	C9—C12	1.532 (5)
O3—C12	1.201 (5)	C10—C11	1.521 (6)
N1—C1	1.403 (5)	C3—H3	0.9300
N1—C8	1.398 (5)	C4—H4	0.9300
N1—C9	1.444 (5)	C5—H5	0.9300
C1—C2	1.478 (6)	C6—H6	0.9300
C2—C3	1.364 (5)	C9—H9	0.9800
C2—C7	1.386 (5)	C10—H10A	0.9700
C3—C4	1.394 (7)	C10—H10B	0.9700
C4—C5	1.383 (7)	C11—H11A	0.9700
C5—C6	1.370 (8)	C11—H11B	0.9700
			
C11—S1—C12	94.86 (18)	S1—C12—O3	125.6 (3)
C1—N1—C8	111.7 (3)	S1—C12—C9	110.3 (2)
C1—N1—C9	125.1 (3)	O3—C12—C9	124.1 (3)
C8—N1—C9	123.0 (3)	C2—C3—H3	121.00
O1—C1—N1	124.6 (4)	C4—C3—H3	122.00
O1—C1—C2	129.6 (4)	C3—C4—H4	119.00
N1—C1—C2	105.7 (3)	C5—C4—H4	119.00
C1—C2—C3	130.5 (4)	C4—C5—H5	119.00
C1—C2—C7	108.2 (3)	C6—C5—H5	119.00
C3—C2—C7	121.3 (4)	C5—C6—H6	121.00
C2—C3—C4	116.9 (4)	C7—C6—H6	121.00
C3—C4—C5	121.4 (4)	N1—C9—H9	107.00
C4—C5—C6	121.3 (5)	C10—C9—H9	107.00
C5—C6—C7	117.2 (4)	C12—C9—H9	107.00
C2—C7—C6	121.9 (4)	C9—C10—H10A	110.00
C2—C7—C8	108.7 (3)	C9—C10—H10B	110.00
C6—C7—C8	129.5 (4)	C11—C10—H10A	110.00
O2—C8—N1	123.4 (4)	C11—C10—H10B	110.00
O2—C8—C7	131.0 (4)	H10A—C10—H10B	108.00
N1—C8—C7	105.6 (3)	S1—C11—H11A	110.00
N1—C9—C10	115.1 (3)	S1—C11—H11B	110.00
N1—C9—C12	111.0 (3)	C10—C11—H11A	110.00
C10—C9—C12	108.3 (3)	C10—C11—H11B	110.00
C9—C10—C11	107.9 (3)	H11A—C11—H11B	109.00
S1—C11—C10	106.4 (2)		
			
C12—S1—C11—C10	−19.3 (3)	C1—C2—C7—C6	−178.6 (3)
C11—S1—C12—O3	−178.8 (4)	C1—C2—C7—C8	−0.4 (4)
C11—S1—C12—C9	−0.9 (3)	C3—C2—C7—C6	−0.3 (6)
C8—N1—C1—O1	−177.2 (3)	C3—C2—C7—C8	178.0 (3)
C8—N1—C1—C2	3.5 (4)	C2—C3—C4—C5	−1.2 (6)
C9—N1—C1—O1	−2.4 (6)	C3—C4—C5—C6	0.5 (6)
C9—N1—C1—C2	178.3 (3)	C4—C5—C6—C7	0.4 (6)
C1—N1—C8—O2	176.8 (3)	C5—C6—C7—C2	−0.5 (5)
C1—N1—C8—C7	−3.8 (4)	C5—C6—C7—C8	−178.3 (4)
C9—N1—C8—O2	1.9 (5)	C2—C7—C8—O2	−178.1 (4)
C9—N1—C8—C7	−178.7 (3)	C2—C7—C8—N1	2.5 (4)
C1—N1—C9—C10	59.6 (4)	C6—C7—C8—O2	−0.1 (7)
C1—N1—C9—C12	−63.9 (4)	C6—C7—C8—N1	−179.5 (4)
C8—N1—C9—C10	−126.2 (3)	N1—C9—C10—C11	−160.7 (3)
C8—N1—C9—C12	110.3 (4)	C12—C9—C10—C11	−35.8 (4)
O1—C1—C2—C3	0.8 (7)	N1—C9—C12—S1	148.7 (2)
O1—C1—C2—C7	178.9 (4)	N1—C9—C12—O3	−33.4 (5)
N1—C1—C2—C3	−180.0 (4)	C10—C9—C12—S1	21.4 (4)
N1—C1—C2—C7	−1.8 (4)	C10—C9—C12—O3	−160.7 (4)
C1—C2—C3—C4	179.0 (4)	C9—C10—C11—S1	34.4 (4)
C7—C2—C3—C4	1.1 (5)		

### Hydrogen-bond geometry (Å, °)

**Table:**

*D*—H···*A*	*D*—H	H···*A*	*D*···*A*	*D*—H···*A*
C10—H10B···O1	0.97	2.52	3.149 (5)	122
